# A Randomized Controlled Trial for Prevention of Postoperative Nausea and Vomiting after Laparoscopic Sleeve Gastrectomy: Aprepitant/Dexamethasone vs. Mirtazapine/Dexamethasone

**DOI:** 10.1155/2022/3541073

**Published:** 2022-04-30

**Authors:** Tarek M. Ashoor, Dina Y. Kassim, Ibrahim M. Esmat

**Affiliations:** ^1^Department of Anesthesia and Intensive Care, Faculty of Medicine, Ain-Shams University, Cairo, Egypt; ^2^Department of Anesthesia and Intensive Care, Faculty of Medicine, Beni-Suef University, Beni-Suef, Egypt

## Abstract

**Background:**

Coadministration of different antiemetics proved to decrease postoperative nausea and vomiting (PONV) after laparoscopic sleeve gastrectomy (LSG). This trial compared aprepitant/dexamethasone (A/D) combination vs mirtazapine/dexamethasone (M/D) combination vs dexamethasone (*D*) alone for prevention of PONV in morbidly obese patients undergoing LSG.

**Methods:**

Ninety patients scheduled for LSG were randomly allocated to receive 8 mg dexamethasone intravenous infusion (IVI) only in the *D* group or in addition to 80 mg aprepitant capsule in the A/D group or in addition to 30 mg mirtazapine tablet in the M/D group. Assessment of PONV was carried out at 0–2 h (early) and 2–24 h (late). The primary outcome was the complete response 0‐24 h after surgery. Collective PONV, postoperative pain, side effects and patient satisfaction score were considered as secondary outcomes.

**Results:**

The A/D and M/D groups were superior to the *D* group for a complete response within 0–24 h after surgery (79.3% for the A/D group, 78.6% for the M/D group, and 20.7% for the *D* group). The *D* group was inferior to the A/D and M/D groups regarding collective PONV and use of rescue antiemetic 0–24 h after surgery (*P* < 0.001, *P* < 0.001, respectively). The peak nausea scores (2–24 h) were significantly reduced in the M/D group in comparison to the *D* group (*P*=0.005). Patients in the M/D group showed high sedation scores, while those in the A/D group showed low pain scores (2–24 h) and less analgesic requirements (*P* < 0.001, *P* < 0.001, *P* < 0.001, respectively). The A/D and M/D groups were superior to the *D* group with regard to the patient satisfaction score (*P* < 0.001).

**Conclusion:**

Aprepitant/dexamethasone combination and mirtazapine/dexamethasone combination were superior to dexamethasone alone in alleviating postoperative nausea and vomiting in morbidly obese patients scheduled to undergo laparoscopic sleeve gastrectomy. Trial Registration: ClinicalTrials.gov identifier: NCT04013386.

## 1. Introduction

Laparoscopic sleeve gastrectomy (LSG) has tremendous growth over the last decade as a treatment of morbid obesity [1]. However, it is associated with postoperative nausea and vomiting (PONV) which agonize nearly 48% of those patients and not only result in distressing patients but also may delay discharge from postanesthesia care unit (PACU) and increase annual cost [2]. This incidence is even increased in female gender, younger age, nonsmoker, history of PONV or motion sickness, increased anesthesia duration, and the use of postoperative opioids [3]. The limited efficacy of a single antiemetic to control PONV after LSG encouraged evaluation of several antiemetic combination strategies [4].

Dexamethasone is a glucocorticoid with a half-life of 36–48 h [5], being used in a dose of 8–10 mg for preventing PONV in patients undergoing chemotherapy and surgery because of its low cost and its limited side effects [6]. It may exert its action through prostaglandin antagonism, prevention of release of serotonins in the gut, and potentiation of other antiemetics by sensitizing the pharmacological receptor [7]. Prophylactic use of dexamethasone in combination with 5-HT_3_ receptor antagonist (5-HT_3_RA) in patients at high risk of PONV was associated with lower use of rescue antiemetics during early or late postoperative periods than dexamethasone or 5-HT_3_RA monotherapy [8].

Aprepitant is a selective neurokinin-1 (NK1) receptor antagonist with a half-life of 9–12 h, and it is active against opioid-induced vomiting. It is used for chemotherapy-induced nausea and vomiting (CINV) and PONV prophylaxis [9, 10]. Aprepitant markedly prevents both acute and delayed emesis in CINV and PONV for the first 24–48 h [9]. The combination of 5-HT_3_RA and aprepitant resulted in a lower incidence of PONV [10].

Mirtazapine is a noradrenergic and specific serotonergic antidepressant (NaSSA) that blocks postsynaptic 5-HT_2_ and 5-HT_3_ receptors. Mirtazapine is effective in controlling CINV and PONV due to blockade of nausea and vomiting receptors [11]. It is rapidly and well absorbed from the gastrointestinal tract after oral administration, and peak plasma concentrations are achieved within 2 hours. The longer elimination half-life of mirtazapine (varying between 20–40 h) justifies a once-daily dosing regimen [12].

The research idea of this study was developed based on patients undergoing bariatric surgeries having many risk factors for PONV; 80% of patients undergoing bariatric surgeries are females [1] (B1 evidence), and most females in our community are nonsmokers (B1 evidence). The gold standard way of surgery is laparoscopic (B1 evidence), the gold standard way of anesthesia is general (A1 evidence), and opioids are used both intra and postoperatively (A1 evidence) [3].

Dexamethasone was selected based on various reviews confirming its antiemetic effects, possible opioid sparing effects in laparoscopic surgeries [6], and it was selected in this study as a positive control, as the authors believed it would be unethical to deny a patient a well-accepted modality of control of nausea and vomiting.

Aprepitant/dexamethasone (A/D) or mirtazapine/dexamethasone (M/D) combinations were selected based on the fact that both drugs (aprepitant and mirtazapine) are available in oral form (easy to give as premedication preoperatively and without the need to put an intravenous (IV) line which is usually difficult in obese patients) and both drugs have the collateral benefit of opioid sparing effects. Aprepitant and mirtazapine could prevent PONV directly and indirectly by reducing the opioids given for postoperative analgesia [9, 11].

It is recommended in the guidelines to combine dexamethasone with aprepitant (A2 evidence) [3]. On the other hand, although mirtazapine was not mentioned by name, combining dexamethasone with 5-HT_3_ blockers was listed in the guidelines [3] and its antiemetic effect was studied in prior studies [11].

The research team conducted this study to test the hypothesis of decreasing the incidence of PONV in morbidly obese patients undergoing LSG by using (A/D) combination vs (M/D) combination vs dexamethasone (*D*) alone and to highlight any associated side effects and patients' satisfaction.

## 2. Methods

This study was performed in Ain-Shams University Hospitals from the 15^th^ of July 2019 till the 31^st^ of December 2019, followed the standards of Helsinki Declaration-2013 and registered at ClinicalTrials.gov (NCT04013386) after approval of the institute ethics committee (FMASU R 36/2019). Every study participant signed a consent. This prospective, randomised, double-blind study included 90 patients, either medically free or with well controlled hypertension and/or diabetes, aged between 25–55 years old, both sexes with body mass index (BMI) ≥35 kg/cm^2^ undergoing LSG. Patients with gastrointestinal disorders, with a history of obstructive sleep apnea, who received an antidepressant or an antiemetic drug within 48 h before surgery or on treatment with systemic glucocorticoids within 4 weeks before surgery were excluded.

Before surgery, patients were instructed to use a visual analog scale (VAS) to rate nausea and pain on a scale of 0 to 10. Patients rated nausea from 0 (no nausea) to 10 (nausea as bad as it could be) and patients rated pain from 0 = no pain to 10 = the worst pain imaginable [13].

According to computer-generated random numbers hidden in sealed opaque envelopes, a nurse got an envelope which decided the patient`s group. Congruent with 1 : 1 : 1 ratio, patients were assigned to three groups (30 each) to obtain the PONV prophylaxis protocol 2 h before surgery and were unaware of their treatment assignment. In the (A/D) group, each patient received 80 mg aprepitant capsule and a placebo tablet (matching mirtazapine) with sips of water and 8 mg dexamethasone intravenous infusion (IVI). In the (M/D) group, each patient received 30 mg mirtazapine tablet and a placebo capsule (matching aprepitant) with sips of water and 8 mg dexamethasone IVI, and in the *D* (positive control) group, each patient received a placebo capsule and a placebo tablet (matching aprepitant and mirtazapine) with sips of water and 8 mg dexamethasone IVI. Dexamethasone was administered diluted in normal saline (sodium chloride 0.9%) infusion over 15 min.

Aprepitant was presented as Emend® capsules, manufactured by Alkermes Pharma Ireland Limited, Athlone, Ireland. Mirtazapine and dexamethasone were presented as Remeron® tablets, N.V. Organon Oss, the Netherlands, and dexamethasone sodium phosphate ampoules (8 mg/2 ml), MUP, Egypt, respectively. The hospital pharmacy prepared the study drugs and then handled them to the ward nurses, who were blinded to the nature of the medications and the patient's group allocation. The placebo was a capsule or tablet of identical size, shape, and color to that of aprepitant or mirtazapine but without the active ingredient. Anesthesia residents who assessed and recorded all data of PONV were unaware of the patient's group assignment.

Patients were prepared by 8 hours fasting before their scheduled operation, and routine aspiration prophylaxis was administered slowly IV over 10 min. Patients were transferred to the operating theater without sedative premedication and were admitted to the operating table in the ramp position. On arrival in the operating room, routine monitoring was applied and venous access was established with two wide-bored cannulas. A central venous catheter was inserted in patients who had difficult venous access. Preparations for difficult airway were in the form of patient preoxygenation with 100% oxygen for 5 min before induction of anesthesia, and a rapid-sequence intubation was carried out with propofol (1.0–1.5 mg/kg) (according to lean body weight) and rocuronium (0.6 mg/kg) (according to ideal body weight), and fentanyl (1.5–2 *μ*g/kg) (according to ideal body weight). Anesthesia was maintained with 1.2–2% sevoflurane in 50% oxygen and air to keep the bispectral index (BIS) value at 40–60 and intermittent doses of muscle relaxant if needed to maintain adequate muscle relaxation throughout the surgery. Mechanical ventilation was maintained by putting patients on a controlled ventilation mode, targeting end-tidal CO_2_ at 35–40 mmHg. During laparoscopy, intra-abdominal pressure was kept up with 12–14 mmHg by carbon dioxide insufflator, and the patient was placed in 20–30° head up position. All surgical procedures were completed by the same surgeon. At the end of surgery, sevoflurane administration was ceased; 0.02 mg/kg atropine and 0.05 mg/kg neostigmine were obtained intravenously for antagonism of neuromuscular blockade. After satisfactory recovery, patients were extubated and transferred to the PACU, where they were monitored with ECG, NIBP, and pulse oximetry, and transferred to the surgical ward when Aldrete's score is more than 9.

Postoperatively, 1 gm of IV paracetamol was given regularly every 8 hours, and 30 mg ketorolac ampoule diluted in 100 ml of normal saline infusion over at least 15 min was administered as a rescue analgesia as requested by the patients. Time to first rescue analgesia (ketorolac) (min) when VAS >3 and the total dose of rescue analgesia (ketorolac) (mg) 0–24 h after surgery were documented. The patient's level of sedation was assessed one hour preoperatively after taking the study drug (before general anesthesia) and at 24 h postoperatively using the Ramsay Sedation Scale (RSS) [14].

The postoperative data (such as vital signs, pain, and sedation scale) were gathered every 4 h. Incidences of early (0–2 h), delayed (2–24 h), and total (0–24 h) PONV were documented by anesthesia residents. Nausea was defined as a subjective distasteful sensation connected with awareness of the urge to vomit, while vomiting was defined as the vigorous expulsion of gastric contents from the mouth.

The severity of postoperative nausea was evaluated using a 4-point verbal descriptive scale (VDS) [15] as follows and in correlation to the VAS nausea score described by the patient: 0–1 (no nausea) (Grade 0), 2–4 mild (Grade I), 5–7 moderate (Grade II), and 8–10 severe (Grade III). If patients experienced intractable nausea for at least 10 min, nausea at a VAS score >4 or more than one emetic episode postoperatively for at least 15 min, they received a rescue dose of 10 mg of metoclopramide intravenously. The primary outcome was the complete response 0–24 h after surgery. Complete response was defined as patients experiencing VAS nausea score ≤4 and no use of rescue therapy 0–24 h after surgery. Secondary outcomes were collective PONV (experiencing nausea and/or vomiting 0–24 h after surgery), postoperative pain and side effects (headache, pruritis, dizziness, somnolence, dry mouth, hypotension, or extra-pyramidal manifestations). Patients' satisfaction with the PONV prophylaxis protocol was assessed 24 h after surgery using a 7-point Likert scale [16] and was also considered as a secondary outcome.

### 2.1. Analysis of Data

Using the PASS 11^th^ release program for sample size calculation [17], the setting power at 80%, the alpha error at 0.017 for three group comparisons [18] and after reviewing results from our pilot study that was carried out to determine the sample size of the current study which included five cases per group, it was found that the rates of complete response 0–24 h after surgery in the A/D, M/D, and *D* groups were 80.0%, 60.0%, and 20.0%, respectively, a sample size of at least 30 patients for each group was needed to reject the null hypothesis of getting statistical differences among the 3 groups.

### 2.2. Statistical Methods

The collected data were coded, tabulated, and statistically analyzed using IBM SPSS statistics (Statistical Package for Social Sciences) software version 22.0, IBM Corp., Chicago, USA, 2013. Descriptive statistics were carried out for quantitative data as mean ± SD (standard deviation), while they were done for qualitative data as number and percentage. Inferential analyses were done for quantitative variables using the Shapiro–Wilk test for normality testing and the ANOVA test. In qualitative data, inferential analyses for independent variables were done using the Chi-square test for differences between proportions and Fisher's exact test for variables with small expected numbers. The post hoc Bonferroni test was used to find homogenous groups in cases of significant differences among the study groups. The *P* value <0.050 was set as a significance cut point.

## 3. Results

**Figure 1:**
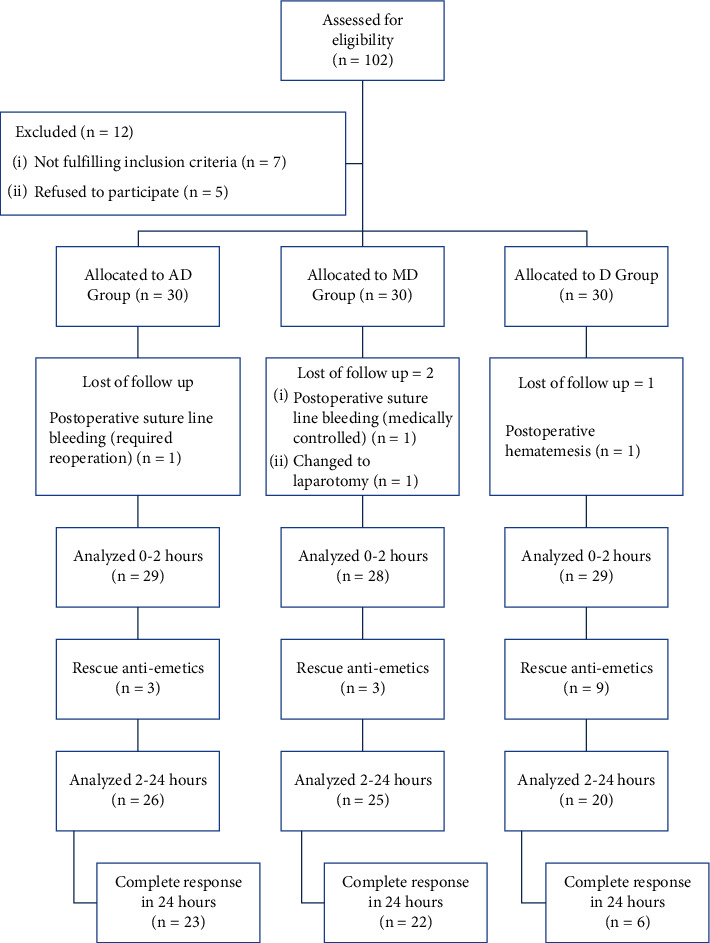
Flow chart of the study. A/D group: aprepitant/dexamethasone group, M/D group: mirtazapine/dexamethasone group, and *D* group: dexamethasone group.

102 patients were screened for eligibility, out of which 90 patients were included who were randomly allocated to the A/D, M/D, and *D* groups. 86 patients completed the study (4 patients were omitted from analysis; 2 patients were re-explored because of bleeding; 1 patient underwent laparotomy due to intraoperative difficulties; and 1 patient developed postoperative hematemesis) (Figure 1). The study groups did not show any significant differences in mean age, BMI, associated comorbidities, history of smoking, history of motion sickness, and/or history of PONV. In concordance to that, the intraoperative variables of duration of surgery and mean given intravascular fluid volume were comparable between groups (Table 1).

In the early postoperative period (0–2 h), there was a statistically significant difference between the A/D group and the *D* group in the number of vomiting episodes (*P*=0.029) with comparable efficacy between the A/D and M/D groups (Table 2). There were no statistically significant differences between the studied groups regarding nausea episodes, collective PONV, rescue antiemetic usage, and the number of patients exhibiting a complete response (Table 2).

**Figure 2:**
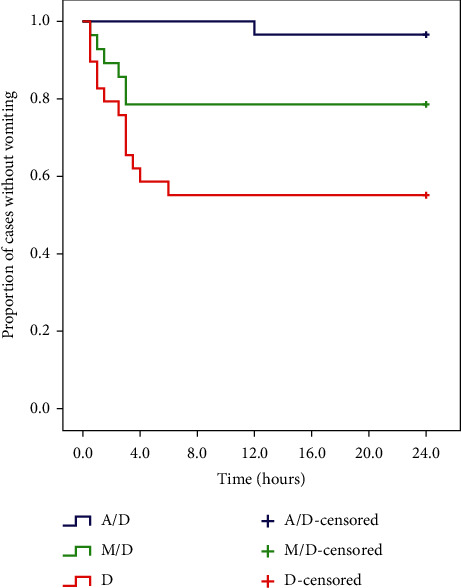
Kaplan–Meier curve for vomiting rate (Log rank test, *P*value <0.001). A/D group: aprepitant/dexamethasone group, M/D group: mirtazapine/dexamethasone group, and *D* group: dexamethasone group.

In the late postoperative period (2–24 h), there was a statistically significant difference between the M/D group and the *D* group regarding the incidence of nausea episodes (*P*=0.005) with comparable efficacy between the A/D and M/D groups (Table 2). The number of vomiting episodes was statistically significantly higher in the *D* group in comparison to the A/D group with comparable efficacy between the A/D and M/D groups (*P*=0.016) (Table 2) (Figure 2). The collective PONV, the rescue antiemetic usage and the proportion of patients exhibiting a complete response were statistically significantly lower in the A/D and M/D groups in comparison to the *D* group with comparable efficacy between the A/D and M/D groups (*P* < 0.001, *P* < 0.001, *P* < 0.001, respectively) (Table 2).

**Figure 3:**
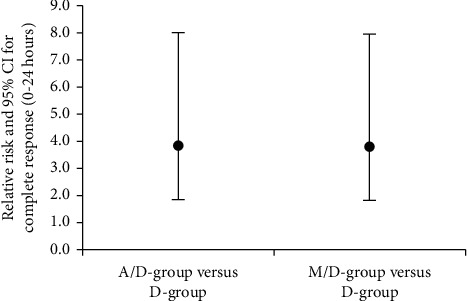
Relative risk with 95% CI for A/D group vs. M/D group for complete response in the first 24 h after surgery; showing that the probability of complete response relative to the *D* group was in the M/D group close to that in the A/D group. A/D group: aprepitant/dexamethasone group, M/D group: mirtazapine/dexamethasone group, and *D* group: dexamethasone group.

For the period (0–24 h), there were statistically significant differences between the M/D group and the *D* group regarding the incidence of nausea episodes and grade of nausea (*P*=0.029, *P*=0.012, respectively) with comparable efficacy between the A/D and M/D groups (Table 2). The number of vomiting episodes was statistically significantly higher in the *D* group in comparison to the A/D group with comparable efficacy between the A/D and M/D groups (*P* < 0.001) (Table 2) (Figure 2). The collective PONV, the rescue antiemetic usage and the proportion of patients exhibiting a complete response were statistically significantly lower in the A/D and M/D groups compared to the *D* group with comparable efficacy between the A/D and M/D groups (*P* < 0.001, *P* < 0.001, *P* < 0.001, respectively) (Table 2). The probability of complete response relative to the *D* group was in the M/D group close to that in the A/D group (Figure 3). Some cases had nausea in the early postoperative period and then vomited later, so the frequency of collective PONV at (0–24 h) after surgery was less than the gross sum of nausea episodes and vomiting episodes alone **(**Table 2).

Preoperative and postoperative sedation scores were significantly higher in the M/D group in comparison to the A/D and *D* groups (*P* < 0.001, *P* < 0.001, respectively) and nonsignificantly higher in the A/D group compared to the *D* group (Table 3).

The pain score at (0–2 h) was significantly higher in the *D* group in comparison to the A/D and M/D groups with comparable efficacy between the A/D and M/D groups (*P* < 0.001). The pain score at (2–24 h) was 2.3 ± 0.5 in the A/D group, 3.8 ± 0.4 in the M/D group, and 4.3 ± 1.1 in the *D* group (*P* < 0.001). The rescue analgesic doses were significantly higher in the *D* group compared to the A/D and M/D groups with statistically significant differences between the A/D and M/D groups (*P* < 0.001) (Table 3).

Incidences of the studied side effects weren't statistically significant among the three groups, and no patient required treatment for adverse effects (Table 3). More patients in the A/D and M/D groups were satisfied with the PONV prevention protocol compared to the *D* group with statistically significant differences between the A/D and M/D groups (*P* < 0.001) (Table 3). No patients needed any specific respiratory care in the postoperative period.

## 4. Discussion

This study demonstrated the favorable response rates with regard to the efficacy of a single dose of 8 mg dexamethasone IVI in combination with 80 mg aprepitant capsule or 30 mg mirtazapine tablet versus dexamethasone alone for prevention of PONV in morbidly obese patients undergoing LSG.

PONV after bariatric surgery can lead to dehydration, electrolyte imbalances, and possibly increase hospital length of stay. A standard regimen has not yet been identified [19]. A multimodal antiemetic approach including 2 or more interventions is recommended [3].

Doses of the aprepitant and dexamethasone combination were based on a previous study which revealed an efficient antiemetic effect in patients at high-risk of PONV from epidural fentanyl analgesia [20]. While doses of mirtazapine and dexamethasone combinations were based on research conducted by Chen et al., who documented that premedication with this combination significantly reduced PONV in patients undergoing gynecological procedures [21]. In addition, the timing and method of administration of preemptive dexamethasone were based on research conducted by De Oliveira et al. [22].

Dexamethasone is recommended as a single perioperative injection of 8–14 mg to decrease PONV in the first 24 h after surgery [23]. It also has analgesic effects, improves respiratory parameters, decreases fatigue, low-cost drug, and promotes better recovery [3, 20, 22, 23]. However, PONV prevention with 8 mg dexamethasone intravenously increased postoperative blood glucose values in nondiabetic and diabetic patients, irrespective of baseline blood glucose levels [24].

NK1 receptor antagonists (e.g., aprepitant) have been shown to be efficient in decreasing postoperative vomiting rather than nausea. This might be due to its deferential affinity for NK1 receptors at peripheral and central levels. It is considered a useful prophylactic antiemetic in bariatric and neurosurgery operations and was found to be effective either alone or in combination to other antiemetic [10, 25]. In concordance with our study, Kakuta et al. concluded that a single preoperative dose of **80** mg aprepitant capsule significantly lowered PONV and pain medications 2–24 h after surgery in patients undergoing laparoscopic gynecological procedures [26]. Sinha et al. documented the efficacy of adding oral **80** mg aprepitant to 4 mg ondansetron intravenously in the reduction of vomiting episodes at 72 h after laparoscopic bariatric surgery [10]. Consistent with our study, Kawano et al. reported that the combination of oral **80** mg aprepitant and 8 mg dexamethasone intravenously had a lower incidence of vomiting episodes from continuous epidural fentanyl analgesia at 24 h after elective knee osteoarthritis surgery [20].

Jeyabalan et al. concluded that a single dose of **40** mg aprepitant was equally effective to 8 mg ondansetron in 3 doses, 8 h apart, in preventing PONV, reducing the severity of nausea and the number of rescue antiemetics during the 24 h postoperative period in 125 women undergoing breast and thyroid surgeries [27]. In addition, Gan et al. demonstrated that aprepitant was superior to ondansetron in patients assigned to obtain a one preemptive dose of oral **40** mg aprepitant or oral **125** mg aprepitant, vs. 4 mg ondansetron intravenously [28]. Over and above, Diemunsch et al. found that aprepitant was noninferior to ondansetron in the prevention of vomiting over 0–24 h and there was no difference in peak nausea scores due to the fact that nausea is subjective and the implementation of different institutional protocols for administration of rescue therapy for nausea and vomiting episodes [29]. In addition, Habib et al. reported that the combination of oral **40** mg aprepitant and 10 mg dexamethasone intravenously was more efficient than the combination of ondansetron and dexamethasone for the prevention of postoperative vomiting in adult patients scheduled for craniotomy under general anesthesia [30].

Partially consistent with this study, Moon et al. documented a decreased nausea severity and increased rescue analgesics in the aprepitant group compared to the palonosetron group, 2 h after administration, in patients scheduled for laparoscopic gynecological surgeries under general anesthesia [13]. Furthermore, in contrast to our study, Bilgen et al. reported that the combination of 8 mg dexamethasone intravenously and oral **40** mg aprepitant did not improve the complete response for PONV in comparison to the combination of 8 mg dexamethasone intravenously and 4 mg ondansetron intravenously in patients undergoing laparoscopic surgery [31].

Results of this study were consistent with the study of Chang et al., who found that a single preoperative oral 30 mg mirtazapine decreased the incidence, delayed the onset and reduced the severity of nausea and vomiting after orthopedic surgery caused by intrathecal morphine in patients scheduled to undergo spinal anesthesia [11]. Also, Omran et al. documented that oral 30 mg mirtazapine premedication significantly reduced preoperative anxiety and the incidence of postoperative early nausea and late vomiting in comparison with 16 mg ondansetron intravenously in 80 female patients undergoing breast surgery [32]. Similarly, Teixeira et al., evaluated the use of oral 30 mg mirtazapine once/day for 2 to 8 months in 2 morbidly obese cases who underwent gastric bypass and experienced nausea and vomiting 1 month after the procedure and reported the disappearance of nausea and vomiting within days after beginning the medication [33].

The combination of dexamethasone with a **5**-HT3 receptor antagonist was advocated for high-risk patients [3, 30, 31]. The reduction of PONV risk in the M/D group compared to the *D* group proved that mirtazapine (with 5-HT_3_ blocking properties) likewise decreased the risk of PONV [11]. Concomitant with our results, Chen et al. found that the combination of mirtazapine plus dexamethasone effectively reduced the level of preoperative anxiety and the frequency of late PONV in gynecological surgeries under general anesthesia [21]. Similarly, Hsu et al. reported that a single oral 30 mg mirtazapine premedication in combination to 8 mg dexamethasone plus 1.25 mg droperidol reduced the incidence of PONV in moderate-to-high-risk patients [34].

Our study confirmed the analgesic effects of aprepitant in the A/D group and mirtazapine in the M/D group compared to dexamethasone alone which were supported with other studies using aprepitant [25, 26] and mirtazapine [35–37]. In contrast to our study, Moon et al. documented increased rescue analgesics in the aprepitant group [13].

Mirtazapine also has the advantage of alleviating preoperative anxiety in a variety of elective surgical procedures [38]. Those results coincided with our results that confirmed the sedative effect of mirtazapine as evidenced by higher Ramsay sedation scores in patients pre-medicated with mirtazapine.

Despite that aprepitant/dexamethasone combination had a significantly better patient satisfaction score than mirtazapine/dexamethasone and dexamethasone alone, yet its high cost may render it not to be cost-effective for PONV prophylaxis. The research team believes that this research will change the clinical practice in PONV prophylaxis in bariatric patients in providing a cheap, available, and effective drug for PONV prophylaxis (mirtazapine). It is not only an oral medication that can be administered easily preoperatively and provides PONV prophylaxis; it also alleviates the patient's anxiety and has an analgesic effect.

This study has many merits. First, the investigators used an evidence-based, low-cost drug and safe antiemetic medication, dexamethasone, as a baseline prophylactic antiemetic [20]. Other study medications, aprepitant and mirtazapine, have oral forms with long-lasting effects up to 24 hours and are cheaper than ondansetron [20]. Together with dexamethasone, they developed additive effects due to their different antiemetic mechanisms of action [3]. Second, the rescue antiemetic used; 10 mg metoclopramide intravenously, has acceptable adverse events and its mechanism of action (*D*_2_ receptor blockade) is different from both the baseline and studied drugs [19]. Third, the randomized and double-blind designs reduced the possibility of bias.

This study, however, had some limitations. First, the baseline risk of PONV was not evaluated due to the usage of dexamethasone as a baseline antiemetic; however, all studied patients were considered high risk for PONV and it would be unethical to deny such high-risk patients any PONV preventive treatment. Second, aprepitant is more expensive than mirtazapine; therefore, a cost-effectiveness analysis of the study drugs was necessary [9, 20]. Third, groups were not matched for comorbidities, and fourthly, the study was conducted in a single center and on a relatively small sample size.

This manuscript was presented as a preprint [39].

## 5. Conclusion

Aprepitant/dexamethasone combination and mirtazapine/dexamethasone combination were superior to dexamethasone alone in alleviating postoperative nausea and vomiting in morbidly obese patients scheduled to undergo laparoscopic sleeve gastrectomy.

## Figures and Tables

**Table 1:** Baseline characteristics among the studied groups.

Variables		A/D group (*N* = 29)	M/D group (*N* = 28)	*D* group (*N* = 29)	*P* value
Age (years)		40.6 (6.1)	39.1 (7.3)	41.5 (6.0)	^^^0.391
BMI (kg.m^−2^)		47.0 (2.1)	46.8 (2.7)	47.2 (2.3)	^^^0.836
Sex, M/F		16/13	17/11	19/10	^#^0.722
Associated comorbidities	Medically free	13	11	13	^#^0.888
	HTN or DM	16	17	16	
Smoking, *n* (%)		12 (41.4)	16 (57.1)	14 (48.3)	^#^0.491
History of motion sickness, *n* (%)		4 (13.8)	2 (7.1)	5 (17.2)	^§^0.609
History of PONV, *n* (%)		3 (10.3)	2 (7.1)	4 (13.8)	^§^0.905
Operation duration (min)		103.1 (7.3)	101.9 (5.4)	102.9 (6.8)	^^^0.761
Intraoperative fluids (ml)		1081.0 (114.5)	1098.2 (76.4)	1070.7 (100.5)	^^^0.571

The values are presented as mean ± SD, number of patients (%), or median (*Q*1, *Q*3). ^ANOVA test. ^#^Chi-square test. ^§^Fisher's exact test. A/D group: aprepitant/dexamethasone group, M/D group: mirtazapine/dexamethasone group, *D* group: dexamethasone group, HTN: hypertension, DM: diabetes mellitus, and PONV: postoperative nausea and vomiting.

**Table 2:** Postoperative nausea and vomiting among the studied groups in the first 24 h.

Variables		A/D group	M/D group	*D* group	*P* value
Number of cases (**0–2** h)		29	28	29	
Number of nausea episodes (%)		4 (13.8)	3 (10.7)	5 (17.2)	^§^0.924
Grade of nausea, *n* (%)	I	0 (0)	1 (33.3)	0 (0)	^§^0.318
	II	1 (25)	2 (66)	2 (40)	
	III	3 (75)	0 (0)	3 (60)	
Number of vomiting episodes (%)		0 (0)^**a**^	3 (10.7)^**ab**^	6 (20.7)^**b**^	^**§**^ **0.029** ^*∗*^
PONV, *n* (%)		4 (13.8)	6 (21.4)	11 (37.9)	^#^0.092
Use of rescue antiemetic, *n* (%)		3 (10.3)	3 (10.7)	9 (31)	^#^0.060
Complete response, *n* (%)		26 (89.7)	25 (89.3)	20 (69)	^#^0.060
Number of cases **(2–24** h**)**		**26**	**25**	**20**	
Number of nausea episodes (%)		6 (23.1)^**ab**^	3 (12.0)^**a**^	11 (55.0)^**b**^	^**#**^ **0.005** ^*∗*^
Grade of nausea, *n* (%)	I	1 (16.7)	2 (66.7)	0 (0)	^§^0.061
	II	3 (50)	1 (33.3)	4 (36.4)	
	III	2 (33.3)	0 (0)	7 (63.6)	
Number of vomiting episodes (%)		1 (3.8)^**a**^	3 (12.0)^**ab**^	7 (35.0)^**b**^	^**§**^ **0.016** ^*∗*^
PONV, *n* (%)		7 (26.9)^**a**^	6 (24)^**a**^	18 (90.0)^**b**^	^**#**^ **<0.001** ^*∗*^
Use of rescue antiemetic, *n* (%)		3 (11.5)^**a**^	3 (12.0)^**a**^	14 (70.0)^**b**^	^**#**^ **<0.001** ^*∗*^
Complete response, *n* (%)		23 (88.8)^**a**^	22 (88)^**a**^	6 (30.0)^**b**^	^**#**^ **<0.001** ^*∗*^
Number of cases (**0–24** h**)**		**29**	**28**	**29**	
Number of nausea episodes (%)		10 (34.5)^**ab**^	6 (21.4)^**a**^	16 (55.2)^**b**^	^**#**^ **0.029** ^*∗*^
Grade of nausea, *n* (%)	I	1 (10)	3 (50)	0 (0)	^**§**^ **0.012** ^*∗*^
	II	4 (40.0)	3 (50)	6 (37.5)	
	III	5 (50)^**ab**^	0 (0)^**a**^	10 (62.5)^b^	
Number of vomiting episodes (%)		1 (3.4)^**a**^	6 (21.4)^**ab**^	13 (44.8)^**b**^	^**#**^ **0.001** ^*∗*^
PONV, *n* (%)		10 (34.5)^**a**^	11 (35.7)^**a**^	27 (93.1)^**b**^	^**#**^ **<0.001** ^*∗*^
Use of rescue antiemetic, *n* (%)		6 (20.7)^**a**^	6 (21.4)^**a**^	23 (79.3)^**b**^	^**#**^ **<0.001** ^*∗*^
Complete response, *n* (%)		23 (79.3)^**a**^	22 (78.6)^**a**^	6 (20.7)^**b**^	^**#**^ **<0.001** ^*∗*^

The values are presented as mean ± SD, number of patients (%), or median (*Q*1, *Q*3). ^#^Chi-square test. ^§^Fisher's exact test. Labels (a, b, c) denotes homogenous groups depending on the post hoc Bonferroni test. ^*∗*^Statistically significant. A/D group: aprepitant/dexamethasone group, M/D group: mirtazapine/dexamethasone group, *D* group: dexamethasone group, and PONV: postoperative nausea and vomiting.

**Table 3:** Postoperative findings among the studied groups.

Variables	A/D group (*N* = 29)	M/D group (*N* = 28)	*D* group (*N* = 29)	*P* value
**Sedation**				
Ramsay sedation scale (preoperative)	1.5 (0.5)^**a**^	2.3 (0.5)^**b**^	1.2 (0.4)^**a**^	^**^**^ **<0.001** ^*∗*^
Ramsay sedation scale (postoperative)	1.7 (0.8)^**a**^	2.9 (0.6)^**b**^	1.3 (0.5)^**a**^	^**^**^ **<0.001** ^*∗*^
**Postoperative pain and analgesia**				
Pain score 0–2 hour	2.1 (0.7)^**a**^	2.2 (0.6)^**a**^	2.8 (0.7)^**b**^	^**^**^ **<0.001** ^*∗*^
Pain score 2–24 hour	2.3 (0.5)^**a**^	3.8 (0.4)^**b**^	4.3 (1.1)^**c**^	^**^**^ **<0.001** ^*∗*^
Time to first rescue analgesia (ketorolac) (min)	89.3 (21)^**a**^	77.9 (17.1)^**b**^	53.4 (13.4)^**c**^	^**^**^ **<0.001** ^*∗*^
Total dose of rescue analgesia (ketorolac), (mg) 0–24 hour	40.7 (17.7)^**a**^	59.3 (15.4)^**b**^	70.3 (15.7)^**c**^	^**^**^ **<0.001** ^*∗*^
**Side effects**				
Headache, *n* (%)	2 (6.9)	2 (7.1)	1 (3.4)	^§^0.867
Dizziness, *n* (%)	3 (10.3)	1 (3.6)	2 (6.9)	^§^0.867
Dry mouth, *n* (%)	1 (3.4)	2 (7.1)	2 (6.9)	^§^0.867
Somnolence, *n* (%)	0 (0)	2 (7.1)	0 (0)	^§^0.103
Diarrhea, *n* (%)	1 (3.4)	0 (0)	0 (0)	^§^1.000
Any side effect, *n* (%)	5 (17.2)	6 (21.4)	3 (10.3)	^§^0.509
**Satisfaction**				
Patient satisfaction score	5.4 (1.6)^**a**^	3.8 (1.4)^**b**^	2.7 (1.3)^**c**^	^**^**^ **<0.001** ^*∗*^

The values are presented as mean ± SD, number of patients (%), or median (*Q*1, *Q*3). ^^^ANOVA test. ^#^Chi-square test. ^§^Fisher's exact test. Labels (a, b, c) denotes homogenous groups depending on the post hoc Bonferroni test. ^*∗*^Statistically significant. A/D group: aprepitant/dexamethasone group, M/D group: mirtazapine/dexamethasone group, and *D* group: dexamethasone group.

## Data Availability

The datasets generated and analyzed during the present study are available from the corresponding author on reasonable request.

## Abbreviations

PONV: Postoperative nausea and vomiting
LSG: Laparoscopic sleeve gastrectomy
5-HT3: 5-hydroxytryptamine receptor type 3
5-HT3RA: 5-HT3 receptor antagonist
NK-1: Neurokinin-1
CINV: Chemotherapy-induced nausea and vomiting
NaSSA: Noradrenergic and specific serotonergic antidepressant
PACU: Postanesthesia care unit
VAS: Visual analogue scale
VDS: Verbal descriptive scale.
